# Environmental temperature influences ophidiomycosis progression and survival in experimentally challenged prairie rattlesnakes (*Crotalus viridis)*

**DOI:** 10.1371/journal.pone.0289641

**Published:** 2023-08-03

**Authors:** Michelle Waligora Kendall, Allison D. Wright, Laura A. Adamovicz, Kennymac Durante, Kirsten E. Andersson, Kelcie Frederickson, Katie Vivirito, Emilie A. Ospina, Martha A. Delaney, Matthew C. Allender

**Affiliations:** 1 Wildlife Epidemiology Lab, College of Veterinary Medicine, University of Illinois Urbana-Champaign, Urbana, Illinois, United States of America; 2 The Veterinary Diagnostic Laboratory, College of Veterinary Medicine, University of Illinois Urbana-Champaign, Urbana, Illinois, United States of America; 3 Zoological Pathology Program, University of Illinois, Brookfield, IL, United States of America; 4 The Brookfield Zoo, Chicago Zoological Society, Brookfield, Illinois, United States of America; Instituto Butantan, BRAZIL

## Abstract

Ophidiomycosis is a prevalent and intermittently pervasive disease of snakes globally caused by the opportunistic fungal pathogen, *Ophidiomyces ophidiicola*. Host response has yet to be fully explored, including the role of temperature in disease progression and hematologic changes. This study enrolled twelve adult prairie rattlesnakes (*Crotalus viridis*) in an experimental challenge with *O*. *ophidiicola* at two temperatures, 26°C (n = 6) and 20°C (n = 6). Each temperature cohort included four inoculated and two control snakes. Assessments involving physical exams, lesion swabbing, and hematology were performed weekly. Differences were observed between inoculated and control snakes in survival, behavior, clinical signs, ultraviolet (UV) fluorescence, hematologic response, and histologic lesions. All inoculated snakes held at 20°C were euthanized prior to study end date due to severity of clinical signs while only one inoculated animal in the 26°C trial met this outcome. In both groups, qPCR positive detection preceded clinical signs with regards to days post inoculation (dpi). However, the earliest appearance of gross lesions occurred later in the 20°C snakes (20 dpi) than the 26°C snakes (13 dpi). Relative leukocytosis was observed in all inoculated snakes and driven by heterophilia in the 20°C snakes, and azurophilia in the 26°C group. Histologically, 20°C snakes had more severe lesions, a lack of appropriate inflammatory response, and unencumbered fungal proliferation and invasion. In contrast, 26°C snakes had marked granulomatous inflammation with encapsulation of fungi and less invasion and dissemination. The results of this study identified that *O*. *ophidiicola*-infected rattlesnakes exposed to lower temperatures have decreased survival and more robust hematologic change, though minimal and ineffective inflammatory response at site of infection. Ophidiomycosis is a complex disease with host, pathogen, and environmental factors influencing disease presentation, progression, and ultimately, survival. This study highlighted the importance of temperature as an element impacting the host response to *O*. *ophidiicola*.

## Introduction

Mycotic infections in wildlife are increasingly prevalent worldwide [1] and occasionally associated with population declines as observed for *Pseudogymnoascus destructans* in bats [2] and *Batrachochytrium dendrobatidis* in amphibians [3]. *Ophidiomyces ophidiicola* is an emergent fungal pathogen that causes the clinical disease ophidiomycosis (formerly Snake Fungal Disease) in snakes [4]. Infection with *O*. *ophidiicola* typically causes dermal lesions such as displaced scales, ulcers, areas of necrosis and dermaitits [5], but involvement of deeper tissues and systemic dissemination are also reported [6]. The lesions associated with *O*. *ophidiicola* have been shown to impact daily functions of infected snakes and cause death [7]. Ophidiomycosis has also been shown to alter the behavior of free-ranging snakes by increasing basking behavior, and altering movement, feeding, and reproduction [8–10]. Ophidiomycosis has been identified in over 60 species of wild snakes worldwide [11], and it is speculated that all snake species are susceptible to infection with *O*. *ophidiicola* [12], however, the risk of disease may vary taxonomically [13]. Since its first report in 2006, *O*. *ophidiicola* has been detected in North America [14], Europe [11], Asia [15], and Australia [16,17]. The true impact of ophidiomycosis on snake conservation is unknown, but it has been associated with declines in one isolated population of timber rattlesnakes (*Crotalus horridus*) in New Hampshire, USA [18]. Ophidiomycosis is also highly prevalent in the last viable population of eastern massasauga rattlesnakes in Illinois [19,20], underscoring the need to understand the impacts of this disease in a variety of hosts and environments.

Hematology is often incorporated into veterinary health assessments of free-ranging and managed populations of reptiles [21–23]. Knowledge of reptile clinical pathology is continually expanding as reference intervals become established [24,25]. However, reptile hematologic response to disease remains poorly characterized [26,27]. Inferences have been made by applying knowledge from canine and feline medicine [26], but these often fall short when dealing with the distinctive qualities of non-mammalian blood. This includes nucleated erythrocytes and thrombocytes, varying dominant leukocyte populations, and cell morphology not documented across other taxa [26–28]. Studies that target specific response variables within an animal’s health panel are required in both field and laboratory settings to define response to external stressors [21,29,30]. Combining such studies with known pathogens allows us to develop our understanding of both host-response and disease pathogenesis [8,31,32].

Prairie rattlesnakes (*Crotalus viridis*) are a North American viperid with a natural history that is largely driven by seasonal temperature changes [33]. Pit vipers have been a focus of study with regards to the emergence of ophidiomycosis [19,34], and prairie rattlesnakes, as crotalids, are considered susceptible to *O*. *ophidiicola*. As ectotherms, snake physiology is dependent on temperature, including immune system function [35,36]. Previous studies in reptiles have shown that clinical signs and immune system activation due to infectious diseases vary substantially in different temperatures [32,37]. Host-response to *O*. *ophidiicola* infection is likely temperature-dependent as well, and while some support for this is present in the literature, the relationship between temperature and disease severity has not yet been examined outside of the context of brumation and/or season [10,38].

The aim of this study is to evaluate the role of temperature in the pathogenesis of *Ophidiomyces* infection in prairie rattlesnakes. We hypothesized that inoculation with *O*. *ophidiicola* would result in ophidiomycosis, and that there would be a difference in timing and severity of disease between snakes maintained at two controlled environmental temperatures as measured by clinical signs, molecular detection, and hematologic response.

## Materials and methods

### Fungus preparation

A pure *Ophidiomyces ophidiicola* isolate (UI-VDL # 12–34933) was cultured from an eastern massasauga (*Sistrurus catenatus*) in 2012 and stored on a potato dextrose agar slant at -80°C. The isolate was propagated on sabaroud dextrose agar at 25°C for 10 days. Conidia were dissociated from the hyphae by vortexing a saline suspension of mycelium then filtering the material through sintered glass wool to remove the hyphae. The number of colony-forming units in the suspension was approximately 10^7^ viable conidia per ml as estimated by hemacytometer count and confirmed by plating serial dilutions.

### Animal procedures and sampling

Twelve adult prairie rattlesnakes (*Crotalus viridis*; 6 males and 6 females), part of a long-term research collection at the University of Illinois, were evenly divided between two 15’9” x 12’ environmental chambers (Rheem Puffer Hubbard, Atlanta, Georgia) maintained at 20°C or 26°C and allowed to acclimate for two weeks. Sample size was determined using the following a priori information: alpha = 0.05, power = 0.8 and an expected difference in disease prevalence of 75% between the inoculated and control groups (75% of the inoculated animals would be infected and 0% of the control animals would be infected within the same trial).

Treatment and control animals were housed singly in 20-gallon enclosures (Neodesha Plastics Inc., Neodesha, Kansas). Temperature experiments were performed simultaneously, such that inoculated and control snakes for each temperature were in the same room, but on opposite sides. Biosecurity included a physical distance (eight feet) between treatment and control snakes delineated by tape on the floor. Foot baths containing 10% bleach were used to enter and leave each room. Disinfection of the floor and equipment contacting a snake (e.g. snake hook, tong, handling tube) was accomplished using a 10% bleach solution with a 60 second contact time [39]. Study personnel entering the room would perform daily assessments and weekly sampling for control snakes first and then inoculated snakes and would not return to the control side of the room without full shower and clothing change. All snakes were bedded on newspaper that was changed when soiled and provided water ad libitum. Pre-killed mice were offered every 1–2 weeks.

Initially, complete physical examinations were performed on day -14 and -7 (day 0 corresponds to date of inoculation), and all snakes were deemed to be free of clinical signs based on criteria previously described [5]. Animals were assessed for overall health with particular attention to lesions consistent with ophidiomycosis [5]. The following data were recorded daily throughout the study: attitude (quiet, alert, responsive [QAR] or bright, alert, responsive [BAR]), behavior (moving, in box, loosely coiled, tightly coiled, or unable to observe in hide box), feces (presence or absence), urates (presence or absence), and skin lesions (presence or absence).

Weekly physical examinations, blood samples, lesion photos, mass, and skin swabs were collected. Venipuncture was performed using a 22- or 25-gauge needle with 1 or 3 mL syringe to collect a whole blood sample from the ventral tail vein. Once drawn, the sample was immediately placed into lithium-heparinized collection tubes (Becton Dickinson and Co., Franklin Lakes, NJ, USA) and inverted multiple times to prevent clotting. Skin swabs were taken using a single swab per snake which was vigorously rubbed over the body surface in eight passes (2 dorsal, 2 ventral, 2 left lateral, 2 right lateral). Body swabs were collected one week prior to inoculation and once weekly for 90 days after challenge. Each swab was placed in a sterile 2.0 ml Eppendorf tube and stored at -20°C until analysis.

Animals were euthanized based on predetermined humane criteria including weight loss of greater than 10% in a one-week period, extreme lethargy, lack of righting reflex, and/or granuloma growth that prevented eating, drinking, or visual activity. Snakes were euthanized by complete anesthesia with ketamine (100 mg//kg) intramuscularly, followed by sodium pentobarbital (390 mg) intravenously. All procedures were approved by the University of Illinois IACUC (Protocol: 19165).

### Animal inoculation and assay methods

Four animals (2 males and 2 females) in each room were randomly selected for experimental challenge using a random number generator (inoculated group) whereas two animals (1 male and 1 female) were maintained as a control (sterile saline-inoculated). An interdermal injection of the *Ophidiomyces* culture (0.1 ml of a pure culture containing 109,000 colony forming units) was administered using a 25-gauge needle and 1 ml syringe (**S1 Fig**). The control (mock-infected) animals were inoculated with a similar volume of sterile saline. All injections were performed over the dorsal mid-body of each snake and specific locations were recorded.

### DNA extraction and *Ophidiomyces ophidiicola* qPCR

DNA extraction and quantitative PCR amplification (qPCR) were performed to detect *O*. *ophidiicola* DNA in swab samples. DNA extraction followed the manufacturer’s recommendations (QIAamp DNA mini Kit, Qiagen Inc., Valencia, CA) with the addition of a one-hour incubation at 37°C with 12.5U of lyticase (Sigma-Aldrich, St. Louis, MO), prior to the lysis step. Following DNA extraction, each sample was assessed for DNA quantity (measured in ng/μl) and quality (using the ratio of absorbance at 260 nm to 280 nm) using spectrophotometry (Nanodrop1000, ThermoFisher Scientific, Wilmington, DE). qPCR was performed using three technical replicates for each sample, standard curve dilution, and non-template control (sterile water) on a QuantStudio3 Real Time PCR system (Applied Biosystems, Foster City, CA) using a previously described protocol [40]. Samples were considered positive if replicates had a mean cycle threshold (Ct) value lower than the lowest detected standard dilution on the same plate. Mean fungal quantities (copies per reaction) were standardized to the total quantity of DNA in the sample by dividing the mean copies/μl for each sample by the DNA concentration, as determined by spectrophotometry, yielding standardized fungal quantities in copies per ng DNA.

### Hematology

Hematology was performed within four hours of sample collection. Packed cell volume (PCV) was determined using sodium heparinized hematocrit tubes (Jorgensen Laboratories, Inc., Loveland, CO 80538) centrifuged at 5,000 rpm for 5 minutes. Total solids (TS) was determined with a hand-held refractometer (Amscope RHC-200ATC refractometer, National Industry Supply, Torrance, CA, USA) using plasma from the hematocrit tubes. Blood films were stained with a modified Wright’s Giemsa stain and one hundred cell leukocyte differential counts were performed under oil immersion (1,000x total magnification) by a single observer (MW). Estimated total leukocyte counts (WBC) were performed by averaging the total number of leukocytes in 10 fields at 400x total magnification, then multiplying this number by 2,000.

### Histopathology

Gross necropsies were performed following euthanasia and tissues were immersion fixed in 10% neutral buffered formalin until processing. Formalin-fixed brain, eye, trachea, lung, esophagus, stomach, splenopancreas, liver, kidney, gonad, oviduct (females), coelomic fat, heart, skin with and without body wall, and vertebral spine (n = 1) were routinely processed and embedded, sectioned at 5 microns, and stained with hematoxylin and eosin for histologic evaluation. Tissues were routinely processed and embedded for histopathology, cut at 3 μm and stained with hematoxylin and eosin. All tissue sections from each snake were evaluated by a board-certified veterinary pathologist (M.A.D.), who was blinded to experimental treatment groups. Skin lesions were graded by severity (none, mild, moderate, severe), predominant process (necrotizing, ulcerative, granulomatous), distribution (focal, multifocal, regionally extensive), and cell/tissue type affected (epidermis/dermis, subcutis, skeletal muscle). Parenchymal organs and other tissues were evaluated for evidence of disseminated infection or additional disease processes. Gomori’s methenamine silver (GMS) staining was performed on all sections of skin to confirm (or exclude) presence of fungal hyphae. A final diagnosis was made based on established criteria, including qPCR detection of Ophidiomyces, presence of intralesional hyaline fungal hyphae, and arthrocondia [5].

### Statistical analyses

General and generalized linear mixed models were constructed to determine differences in multiple behavioral, physiologic, and infection parameters based on temperature, treatment group, and study day (R version 4.0.2; R Core Team, 2022; packages lme4, lmerTest, [41–43]). Categorical dependent variables assessed using generalized linear mixed models included attitude (QAR vs. BAR), behavior (in hidebox, moving, loosely coiled, tightly coiled, straight), urination (present vs. absent), defecation (present vs. absent), skin shedding (yes vs. no), appetite (ate vs. did not eat), skin lesions (present vs. absent), skin fluorescence under UV light (present vs. absent), and *O*. *ophidiicola* qPCR result (positive vs. negative). Continuous dependent variables assessed using general linear mixed models included body mass, *O*. *ophidiicola* qPCR quantity, and hematologic parameters. Independent variables for each of these models included study day (-14 through 90 days post-inoculation), temperature (20°C vs. 26°C), and treatment group (inoculated vs. control) as fixed effects, and snake ID as a random effect. Interaction between study day, temperature, and treatment was tested within each model, and if non-significant, then additive effects were considered alone. Post-hoc testing was performed using the emmeans package [44] with a Tukey correction to control for multiple statistical comparisons [44]. Contrasts for continuous dependent variables were specifically considered at inoculation (day 0) and at the last sample date that all snakes remained alive (day 49). Figures were constructed using the ggeffects package [45].

Life tables were constructed for days to death. Individuals that survived to the completion of the study were censored at day 90 as were apparently healthy control animals in the 20°C group that were euthanized on day 69. Kaplan-Meier estimates were used to determine any difference in survival based on inoculation status and environmental temperature (packages survival [46,47] and survminer [48]).

Additional modeling was performed to evaluate associations between lesion characteristics and diagnostic test results, and to determine agreement between diagnostics for *O*. *ophidiicola* infection. The effects of lesion type (none, displaced scale, crust, granuloma, ulcer, multiple) and number of skin lesions (independent variables) on DNA concentration, *O*. *ophidiicola* qPCR quantity, and hematologic parameters (dependent variables) were modeled using general linear mixed models with snake ID as a random effect. The effect of DNA concentration (independent variable) on *O*. *ophidiicola* qPCR result (positive vs. negative; dependent variable) was assessed using generalized linear mixed models with snake ID as a random effect. Agreement between the presence of skin lesions, UV fluorescence, and *O*. *ophidiicola* qPCR result was assessed using Cohen’s kappa, with interpretation following [49]. Receiver operating curves (ROC) were used to create cutoff values and determine the sensitivity and specificity of these cutoff values for predicting the presence of skin lesions and UV fluorescence based on *O*. *ophidiicola* copy number (package pROC, [50]).

## Results

### Survival

A single snake in the 26°C inoculated group died in the first two weeks of the trial due to an unrelated illness (bacterial sepsis) and was removed from all further statistical analysis. All inoculated snakes in the 20°C trial were euthanized due to the severity of clinical signs, while only one (33%) inoculated animal was euthanized in the 26°C trial due to severity of clinical signs (on day 62). The remaining two inoculated animals in the 26°C trial survived with mild-moderate clinical signs and were euthanized at the termination of the study (90 dpi). All control animals survived throughout the study period (69 days for the 20°C group and 90 days for the 26°C group) and were censored.

**Fig 1:**
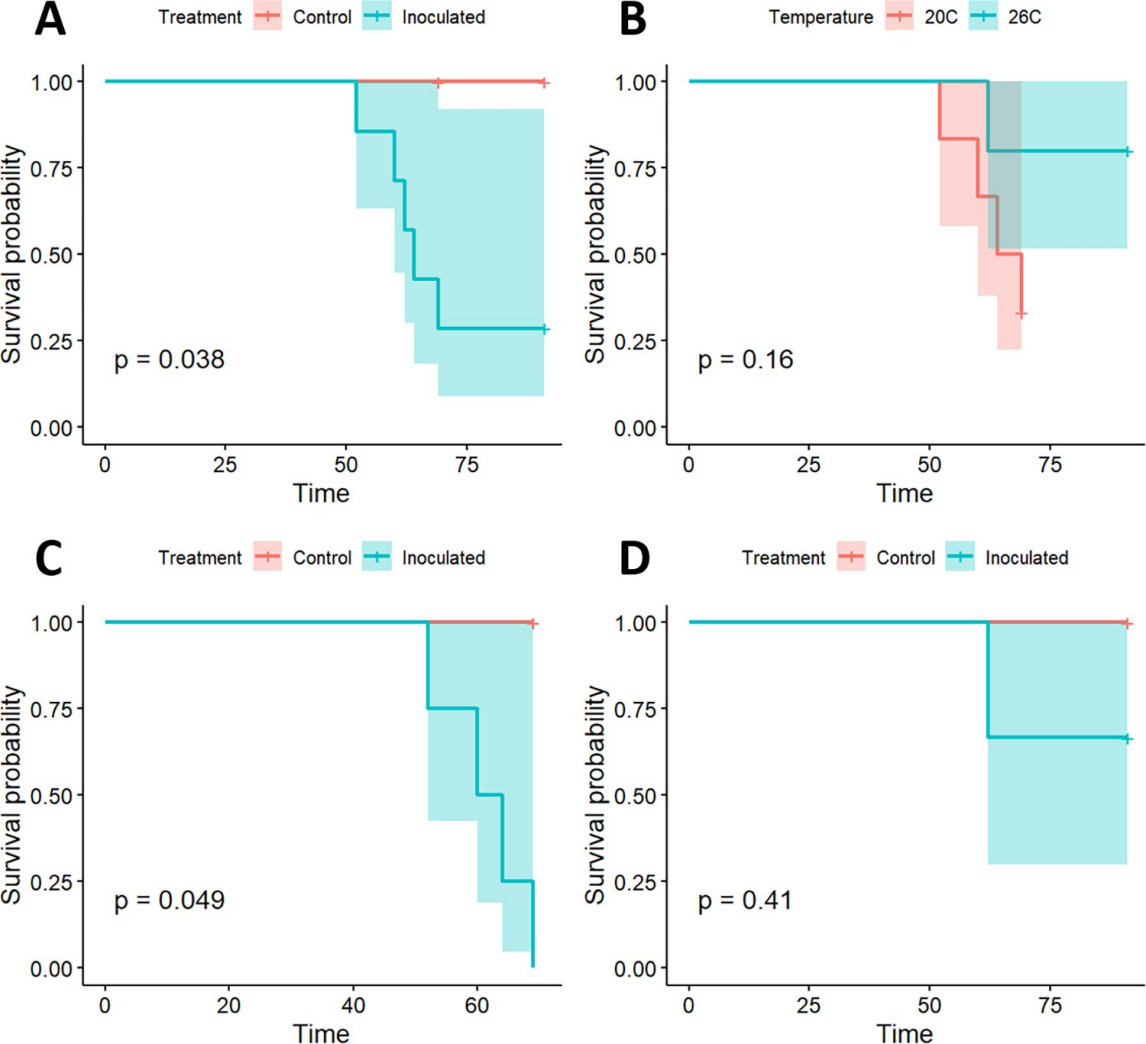
Survival of prairie rattlesnakes inoculated with Ophidiomyces. Survival curves for prairie rattlesnakes (*Crotalus viridis*) experimentally inoculated with *Ophidiomyces ophidiicola* or sterile saline and held at two temperatures (20°C or 26°C). A) Assessment of survival for all snakes based on treatment. B) Assessment of survival for all snakes based on temperature. C) Assessment of survival for treatment and control snakes at 20°C. D) Assessment of survival for treatment and control snakes at 26°C.

When all snakes were included in survival analysis, treatment was a significant predictor of survival (p = 0.038), with a median survival time (MST) of 64 days in the inoculated group and all observations in the control group censored at 69 or 90 days, as appropriate (Fig 1A). Temperature was not a significant predictor of survival when all snakes were included (p = 0.16; Fig 1B). Treatment was a significant predictor of survival (p = 0.049) when the 20°C group was considered separately, with an MST of 62 days in the inoculated group (Fig 1C). However, treatment was not a significant predictor of survival when the 26°C group was considered in isolation (p = 0.41, Fig 1D).

### Behavioral and physiologic parameters

Multiple behavioral and physiologic parameters were impacted by temperature and treatment. The odds of consuming offered meals were 9.97 times higher at 26°C vs. 20°C (95% CI = 1.4–71, p = 0.02). Similarly, the odds of defecation were 1.83 times higher at 26°C vs. 20°C (95% CI = 1.05–3.18, p = 0.03) and the odds of shedding were 31.8 times higher at 26°C vs. 20°C (95% CI = 1.97–512, p = 0.01). The odds of occupying the hidebox were 5.21 times higher for control vs. inoculated snakes (95% CI = 1.36–20, p = 0.02) and were 9.25 times higher for snakes at 20°C vs. 26°C (95% CI = 2.54–33.7, p = 0.0007). The odds of being observed moving were 2.3 times higher for inoculated vs. control snakes (95% CI = 1.19–4.46, p = 0.01) and were 6.08 times higher for snakes at 26°C vs. 20°C (95% CI = 3.21–11.5, p < 0.0001). The odds of being loosely coiled were 3.66 times higher in snakes at 26°C vs 20°C (95% CI = 1.46–9.16, p = 0.006).

### Clinical signs

All snakes were free of skin lesions for over 24 months prior to inoculation with *O*. *ophidiicola* or saline injection. Skin lesions consistent with ophidiomycosis developed in all *O*. *ophidiicola* inoculated snakes at both temperatures, with the first lesion appearing at the site of inoculation at a median of 20 days post inoculation (dpi) in the 20°C group (range 13–27 dpi) and 13 dpi (range 6–20 dpi) in the 26°C group. Initial lesion categories included displaced scales (N = 3), crusts (N = 2), ulcers (N = 1), and granulomas (N = 1), though these lesions frequently progressed to different categories over time. Specifically, two displaced scales became a crust, one displaced scale became a necrotic scale then a crust, and both crusts and the granuloma became ulcers. Three inoculated snakes had only one lesion throughout the study, one had two lesions, two developed three lesions, and one snake had four lesions. These additional lesions included two displaced scales, a necrotic scale, an ulcer, a crust, a displaced scale that progressed to an ulcer, a displaced scale that progressed to a crust then a necrotic scale, and a necrotic scale that progressed to an ulcer.

Neither control snake in the 26°C group developed skin lesions, but both snakes in the 20°C group developed a lesion consistent with ophidiomycosis. This included a displaced scale that developed 27 dpi in one snake, and a displaced scale that progressed to an ulcer at 41 dpi in the second snake. Both of these snakes tested qPCR negative for *O*. *ophidiicola* for the duration of the study.

**Fig 2:**
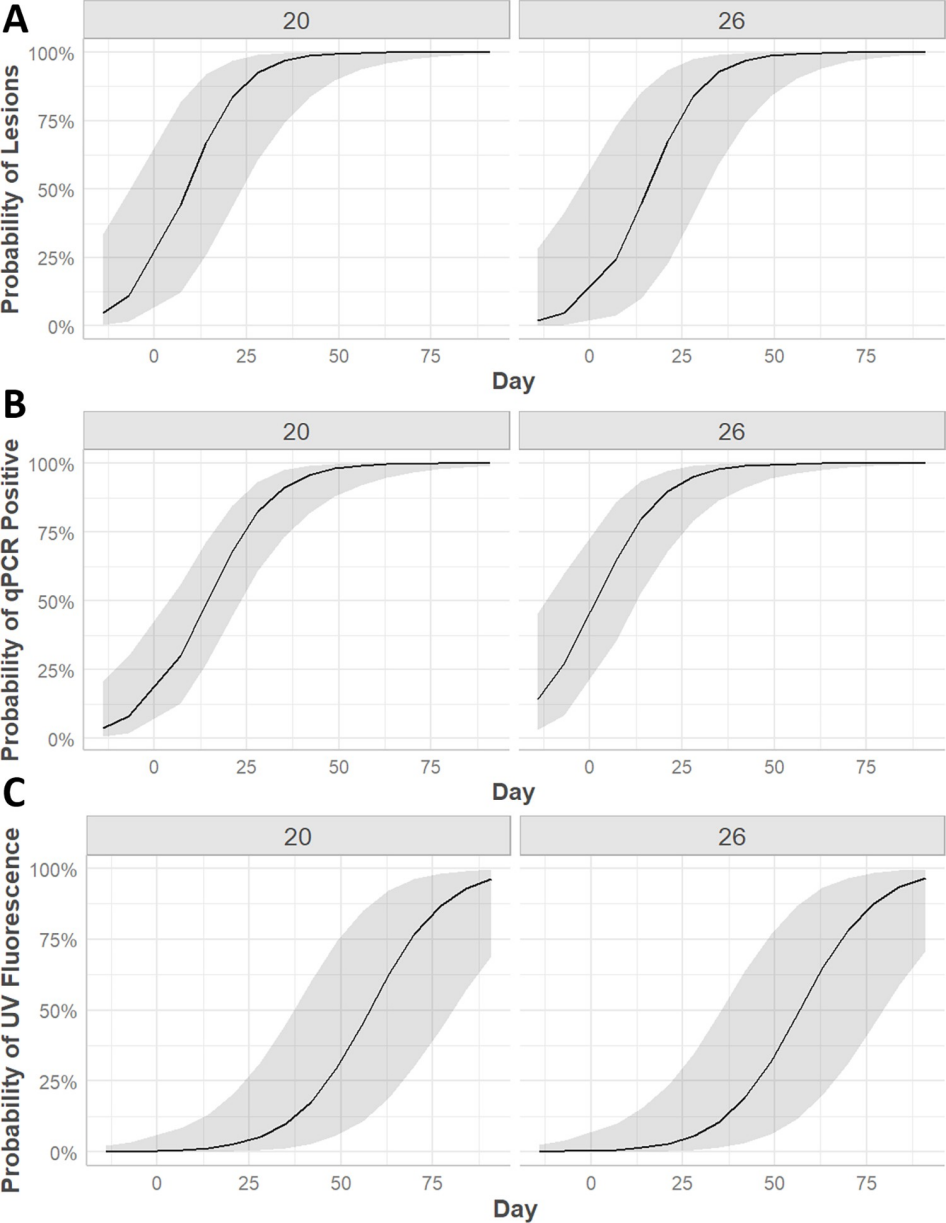
Timing of binomial probability of developing clinical signs, qPCR positivity, and UV fluorescence. The probability of prairie rattlesnakes (*Crotalus viridis*) inoculated intradermally with *Ophidiomyces ophidiicola* developing skin lesions (A), qPCR positivity for *O*. *ophidiicola* (B), and skin fluorescence under ultraviolet light (C) over time. Figures were generated using general linear mixed models with study day and environmental temperature (20°C or 26°C) as fixed effects and snake ID as a random effect. Study day was significantly associated with all dependent variables (p < 0.05), but environmental temperature was not (p > 0.05).

The odds of skin lesion presence were significantly associated with study day and treatment, but not temperature (Fig 2A). Specifically, the odds of developing a skin lesion were 39.5 times higher in the inoculated vs. control groups (95% CI = 11.2–139, p < 0.0001) and elevated 1.15 times for every one additional day post-inoculation (95% CI = 1.09–1.25, p < 0.0001). Number of skin lesions and lesion type were not significantly different between the treatment and temperature groups (p > 0.05).

### Molecular associations and *Ophidiomyces ophidiicola* qPCR

Body swabs from all snakes tested negative for *O*. *ophidiicola* on days -14 and -7. Control snakes at both temperatures tested negative for the duration of the study. All inoculated snakes became qPCR positive for *O*. *ophidiicola* seven days post-inoculation except for one snake in the 20°C group, which was subsequently positive on the next testing day (14 dpi). Like lesion development, the odds of qPCR positivity were significantly associated with study day and treatment, but not temperature (Fig 1B). The odds of testing qPCR positive for *O*. *ophidiicola* were 282 times higher in the inoculated vs. control groups (95% CI = 16.3–4,898, p = 0.0001) and rose 1.1 times with every additional day post-inoculation (95% CI = 1.06–1.17, p < 0.0001).

**Fig 3:**
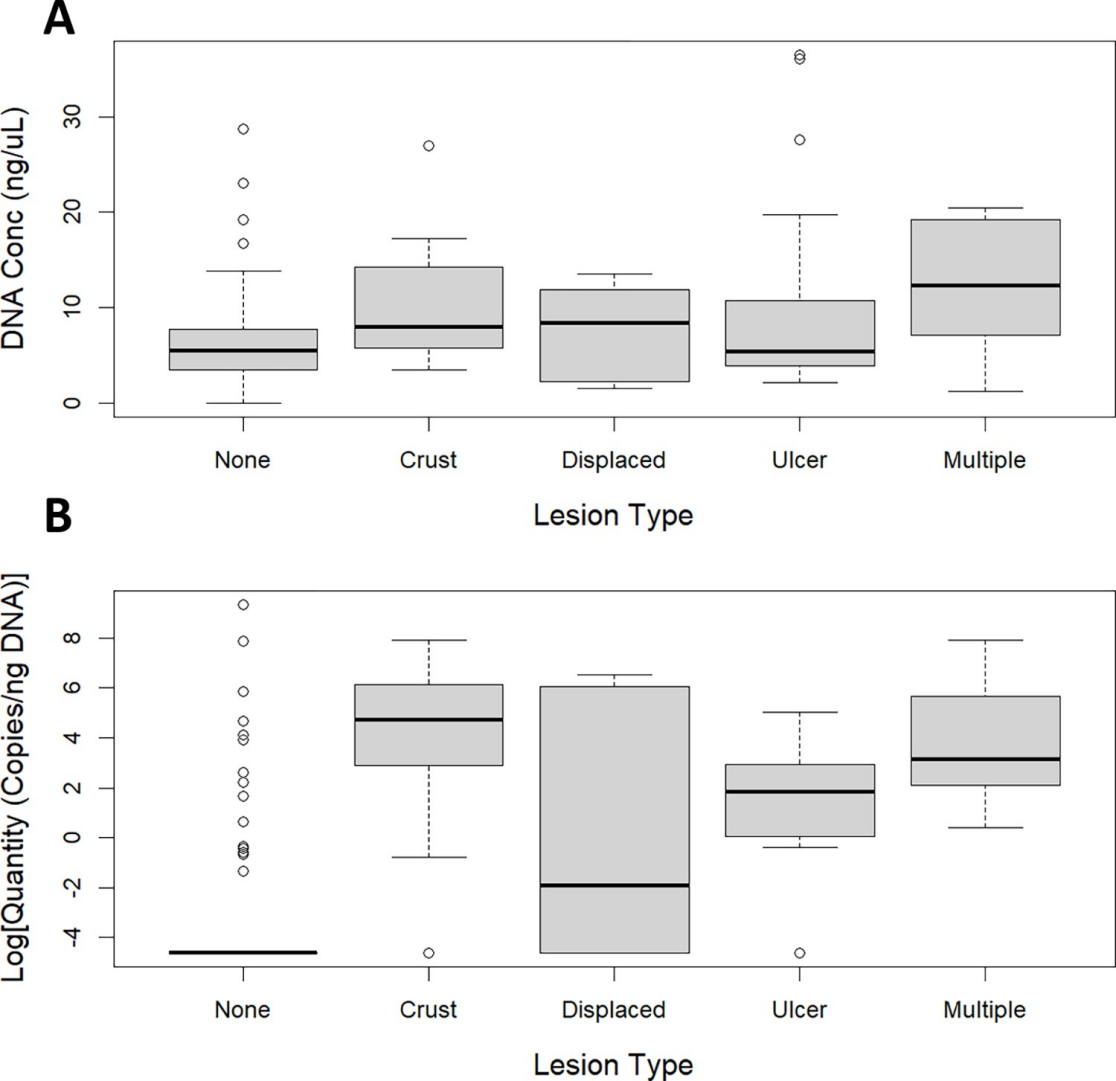
DNA concentration by lesion type. DNA concentration (A) and *Ophidiomyces ophidiicola* qPCR quantity (B) for body swabs collected from prairie rattlesnakes (*Crotalus viridis*) experimentally inoculated with *O*. *ophidiicola* or sterile saline, based on the type of skin lesion present. Displaced = displaced scale, multiple = more than one lesion type present.

Body swab DNA concentration was not significantly associated with temperature (p = 0.96), treatment (p = 0.94), *O*. *ophidiicola* qPCR result (p = 0.33), or lesion type (p = 0.23; Fig 3A), however, there was a significant positive association between DNA concentration and number of skin lesions (p < 0.0001).

*O*. *ophidiicola* qPCR quantities were significantly greater in inoculated vs. control snakes (p < 0.0001), greater in snakes with skin lesions vs. those with none (p < 0.0001), and were positively associated with the number of skin lesions present (p < 0.0001). Body swab fungal quantities were also associated with lesion type (p < 0.0001; Fig 3B), with higher quantities in snakes with crusts (effect size = 676 copies, 95% CI = 61–7,473 copies, p < 0.0001), ulcers (effect size = 113 copies, 95% CI = 11–1,120 copies, p < 0.0001), and multiple co-occurring lesion types (effect size = 1,074 copies, 95% CI = 52–21,982 copies, p < 0.0001) compared to body swab quantities from snakes with no skin lesions. Body swab fungal copy numbers were not significantly associated with temperature (p = 0.78).

### Skin fluorescence under ultraviolet light

**Fig 4:**
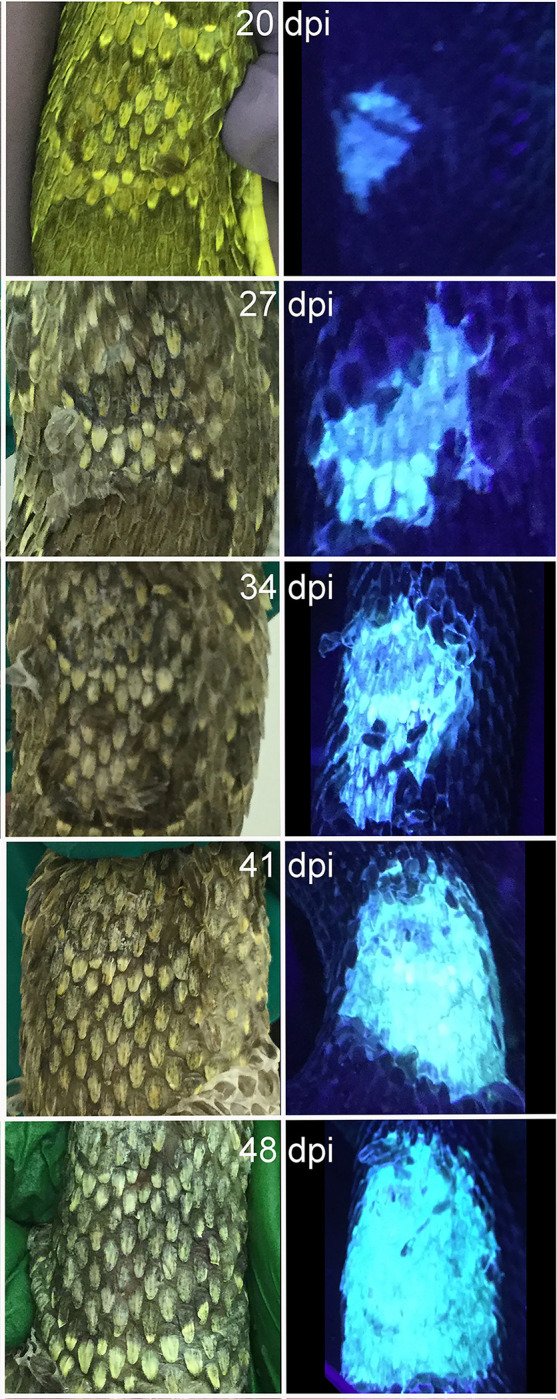
Macroscopic and UV fluorescence of Ophidiomyces in Prairie rattlesnakes. Photograph (left column) and ultraviolet fluorescence (right column) of the dorsum of the same area of an individual prairie rattlesnake (*Crotalus viridis*) over time following experimental inoculation with *Ophidiomyces ophidiicola*. the same area of an individual prairie rattlesnake (*Crotalus viridis*) over time following experimental inoculation with *Ophidiomyces ophidiicola*.

Skin fluorescence under UV light was not present in any snake prior to inoculation. Post-inoculation, fluorescence was present in all inoculated snakes and three out of four control snakes. The median day to first observation of fluorescence was 21 dpi for 20°C inoculated snakes (range 14–42 dpi) and 21 dpi for 26°C inoculated snakes (range 7–35 dpi). Fluorescence was first observed on days 21 and 28 dpi for 20°C control snakes, and 28 dpi for 26°C control snakes. Once UV skin fluorescence was present in an inoculated snake, it remained present for all subsequent assessments (Fig 4). In contrast, the presence of fluorescence was intermittent in control snakes.

The odds of skin fluorescence being present were 47.8 times higher in inoculated vs. control snakes (95% CI = 4.3–532, p = 0.002) and increased 1.1 times with every additional study day (95% CI = 1.07–1.15, p < 0.0001), however, the association between skin fluorescence and temperature was not significant (p = 0.92) (Fig 2). The odds of skin fluorescence were 66.9 times higher in snakes with skin lesions vs. those without (95% CI = 14.4–311, p < 0.0001), and the odds increased 56 times for every additional skin lesion present (95% CI = 14.6–374, p < 0.0001). The odds of skin fluorescence were higher in snakes with crusts (OR = 59, 95% CI = 2.7–1,246, p = 0.003), ulcers (OR = 29, 95% CI = 2.6–322, p = 0.001), and multiple concurrent lesion types (OR = 12, 95% CI = 1.05–143, p = 0.04) compared to those with no skin lesions. Finally, the odds of skin fluorescence were 15.3 times higher in snakes testing qPCR positive for *O*. *ophidiicola* (95% CI = 5.41–43.2, p < 0.0001).

### Agreement between *Ophidiomyces ophidiicola* diagnostics

Cohen’s kappa was used to assess agreement between the presence of skin lesions, qPCR positivity for *O*. *ophidiicola*, and presence of skin fluorescence under UV light. Strong agreement was present between lesion presence and qPCR result (kappa = 0.804, 95% CI = 0.703–0.905), moderate agreement was present between lesion presence and UV fluorescence (kappa = 0.703; 95% CI = 0.58–0.826), and weak agreement was documented between UV fluorescence and qPCR result (kappa = 0.563; 95% CI = 0.419–0.706).

**Fig 5:**
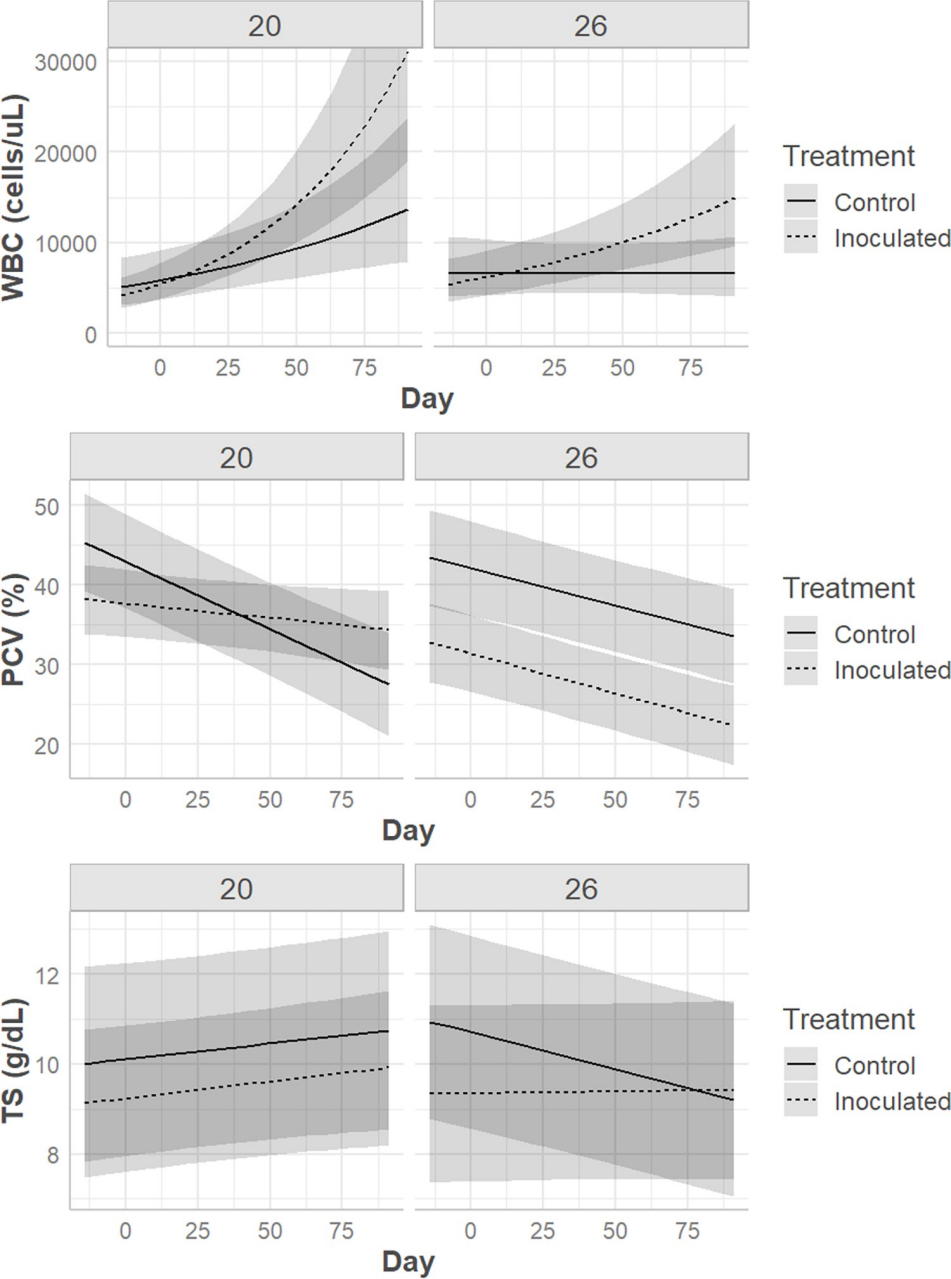
Receiver operating curves. Receiver operating curves for A) Normalized *Ophidiomyces ophidiicola* qPCR copy number (copies/ng DNA) predicting the presence of skin lesions in prairie rattlesnakes (*Crotalus viridis*) experimentally inoculated with *O ophidiicola*. B) Normalized *O*. *ophidiicola* qPCR copy number (copies/ng DNA) predicting skin fluorescence under UV light.

*O*. *ophidiicola* qPCR quantity was a statistically significant predictor of both the presence of skin lesions (p < 0.0001) and the presence of skin fluorescence under UV light (p = 0.03). A cutoff value of 0.345 *O*. *ophidiicola* target copies/ng DNA is 88.5% sensitive and 93.1% specific for predicting the presence of skin lesions, with an associated area under the receiver operating curve (AUC) of 0.927 (Fig 5, Table 1). This same cutoff is 75% sensitive and 82.8% specific for predicting the presence of UV fluorescence, with an AUC of 0.795.

**Table 1:** Diagnostic characteristics of *Ophidiomyces ophidiicola* qPCR copy number cutoff values for predicting the presence of skin lesions and the presence of skin fluorescence under ultraviolet light in prairie rattlesnakes (*Crotalus viridis*) experimentally inoculated with *O ophidiicola*.

Parameter	Outcome	Results	Unit
Area Under the Curve	Lesion Presence	0.927	Copies/ng DNA
Cutoff (copies/ng DNA)	Lesion Presence	0.345	Copies/ng DNA
Sensitivity	Lesion Presence	88.5	%
Specificity	Lesion Presence	93.1	%
Accuracy	Lesion Presence	91	%
True Negative	Lesion Presence	68	Samples
True Positive	Lesion Presence	54	Samples
False Negative	Lesion Presence	7	Samples
False Positive	Lesion Presence	5	Samples
Negative Predictive Value	Lesion Presence	90.7	%
Positive Predictive Value	Lesion Presence	91.5	%
Area Under the Curve	UV Fluorescence	0.795	Copies/ng DNA
Cutoff (copies/ng DNA)	UV Fluorescence	0.345	Copies/ng DNA
Sensitivity	UV Fluorescence	75	%
Specificity	UV Fluorescence	82.8	%
Accuracy	UV Fluorescence	78.9	%
True Negative	UV Fluorescence	53	Samples
True Positive	UV Fluorescence	48	Samples
False Negative	UV Fluorescence	16	Samples
False Positive	UV Fluorescence	11	Samples
Negative Predictive Value	UV Fluorescence	76.8	%
Positive Predictive Value	UV Fluorescence	81.4	%

### Hematology

**Fig 6:**
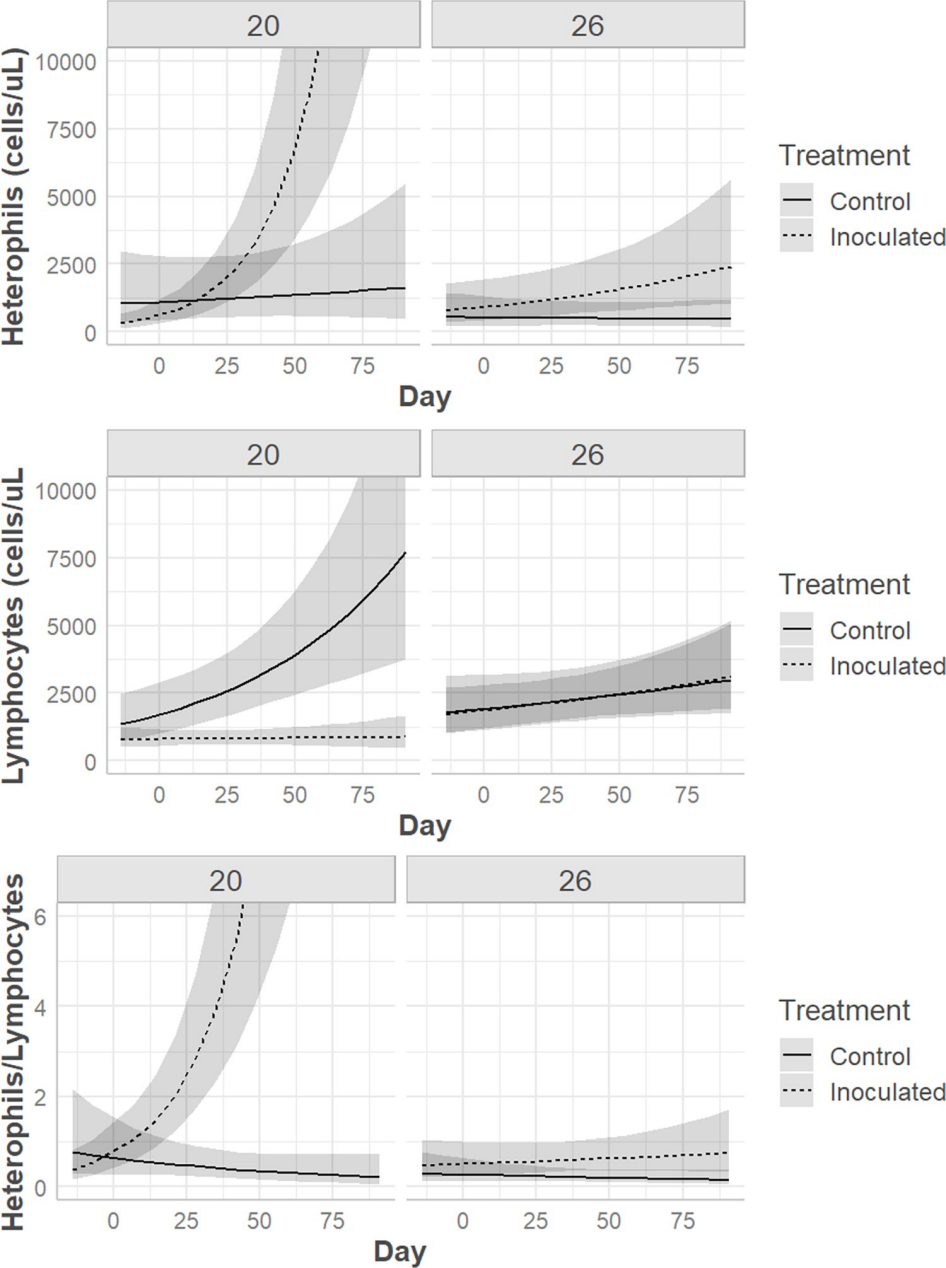
Hematologic response in Prairie rattlesnakes inoculated with Ophidiomyces. Changes in total leukocyte count (WBC), packed cell volume (PCV), and total solids (TS) in prairie rattlesnakes (*Crotalus viridis*) either inoculated with *Ophidiomyces ophidiicola* (“Inoculated”) or injected with sterile saline (“Control”) at two different ambient temperatures (20°C vs. 26°C) over time.

**Fig 7:**
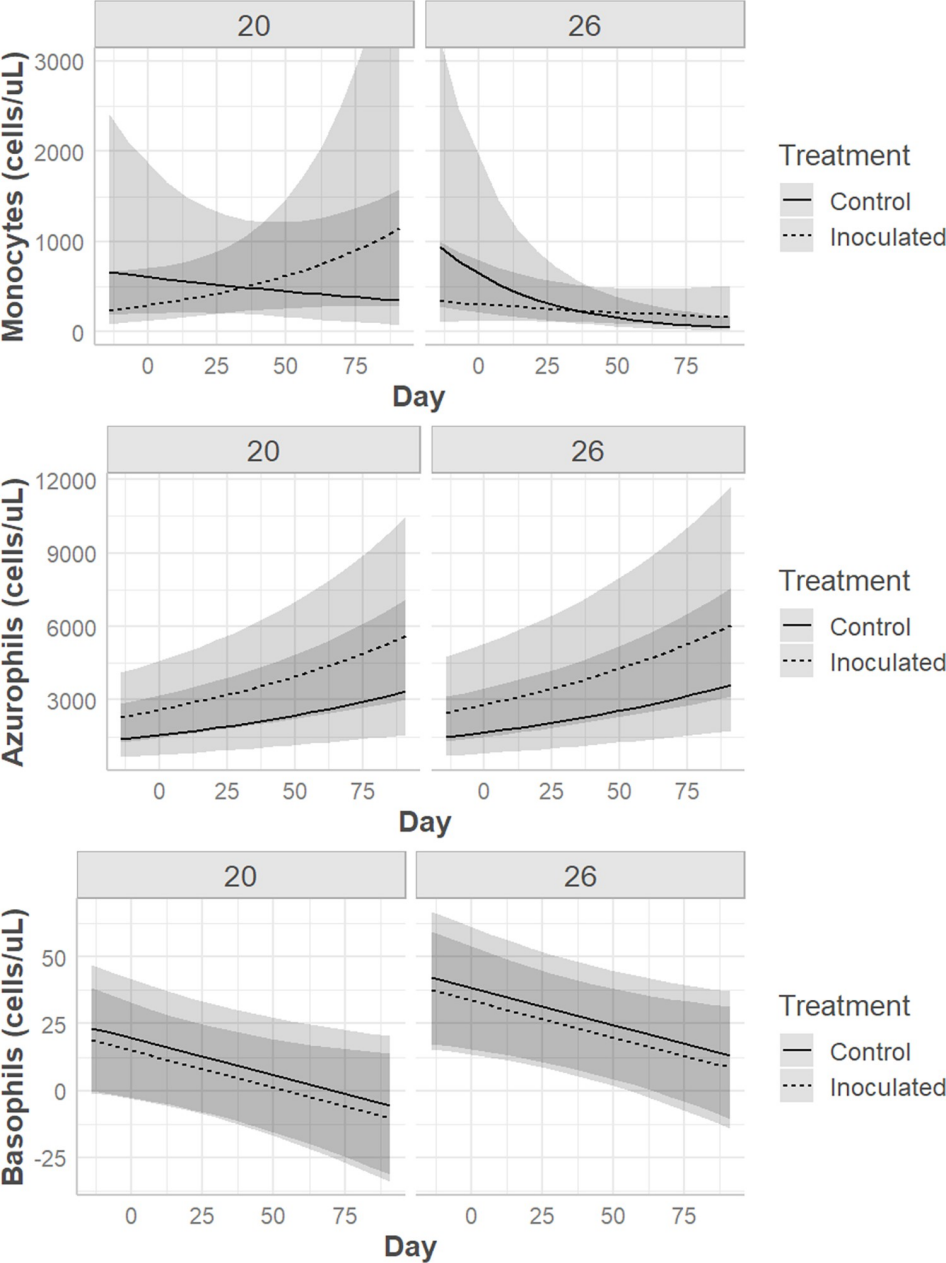
Hematologic response in Prairie rattlesnakes inoculated with Ophidiomyces. Changes in heterophil count, lymphocyte count, and heterophil:lymphocyte ratio in prairie rattlesnakes (*Crotalus viridis*) either inoculated with *Ophidiomyces ophidiicola* (“Inoculated”) or injected with sterile saline (“Control”) at two different ambient temperatures (20°C vs. 26°C) over time.

**Fig 8:**
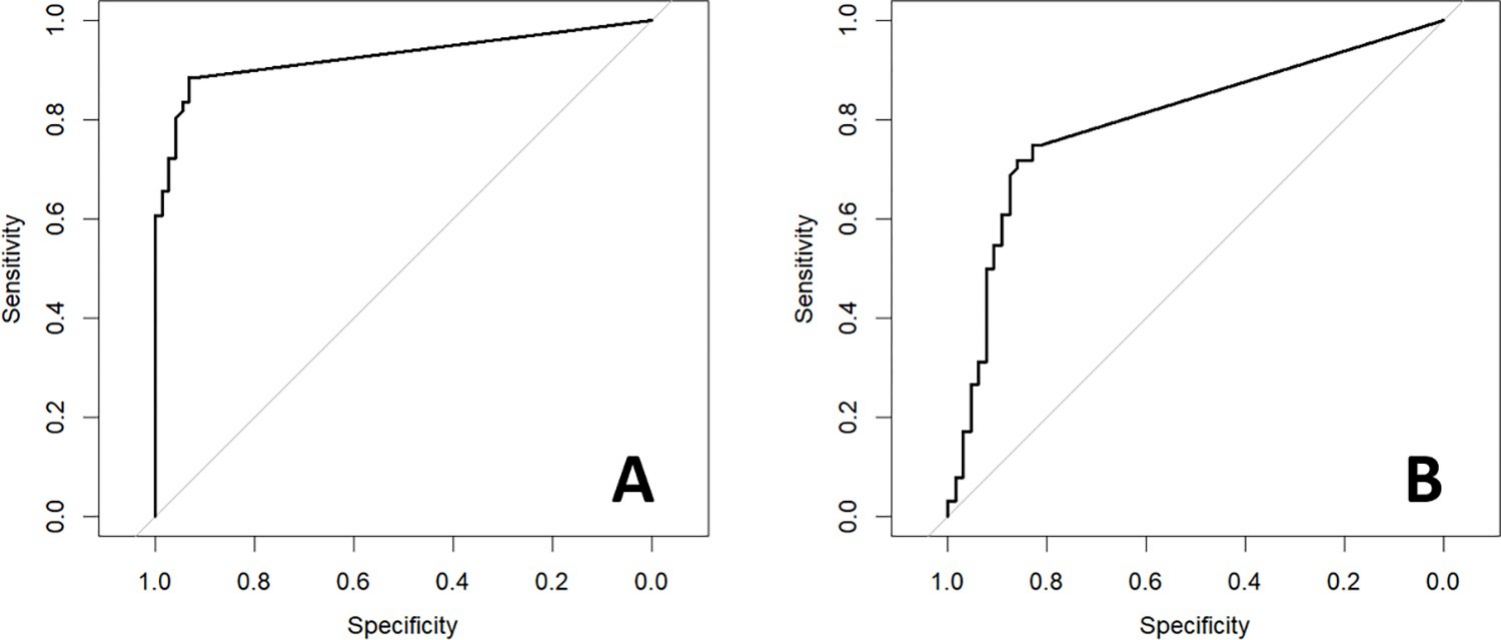
Hematologic response in Prairie rattlesnakes inoculated with Ophidiomyces. Changes in monocyte count, azurophil count, and basophil count in prairie rattlesnakes (*Crotalus viridis*) either inoculated with *Ophidiomyces ophidiicola* (“Inoculated”) or injected with sterile saline (“Control”) at two different ambient temperatures (20°C vs. 26°C) over time.

Prior to and on the day of inoculation there were no statistically significant hematologic differences between treatments or temperatures. Following inoculation, multivariable models revealed that the drivers of hematologic values were frequently complex. A three-way significant interaction effect was noted between day, temperature, and treatment for PCV (p = 0.0008), TS (p = 0.04), heterophil count (p = 0.02), lymphocyte count, (p = 0.04) and the heterophil:lymphocyte ratio (H:L; p = 0.001) (Figs 6 & 7). WBC and monocyte count were significantly associated with two-way interactions between day and temperature (WBC p = 0.005, monocyte p = 0.04) and day and treatment (WBC p = 0.002, monocyte p = 0.04) (Figs 6 and 8). Azurophils (p < 0.0001) and basophils (p = 0.04) were associated with study day (p < 0.0001) (Fig 7).

From day 0 to day 49 pi control snakes had significant declines in PCV at both temperatures, while only snakes at 26°C had corresponding decreases in TS (Table 2). Control snakes also had increases in azurophil count at both temperatures (Table 2). Controls at 20°C had significant increases in lymphocyte count while those at 26°C remained stable (Table 2).

**Table 2:** Differences in control prairie rattlesnake (*Crotalus viridis*) hematologic parameters between study day 49 (last day all snakes remained alive) and study day 0 (date of inoculation with *Ophidiomyces ophidiicola* or sterile saline injection) at two different ambient temperatures. Day 0 is the reference value.

**Analyte**	**Units**	**Temp (** ^**o**^ **C)**	**Mean Study Day 0**	**Mean Study Day 49**	**Difference**	**95% Confidence Interval**	**P-value**
**PCV**	**%**	20	42.7	34.6	-8.0	(-11.6, -4.5)	< 0.0001
		25.6	41.9	37.5	-4.5	(-7.1, -1.8)	< 0.0001
**TS**	**g/dL**	20	8.8	9.1	0.3	(-0.4, 1.1)	0.8
		25.6	9.3	8.5	-0.8	(-1.3, -0.3)	0.0004
**WBC**	**/μL**	20	6,831	10,737	3,906	(-1,137, 8,948)	0.3
		25.6	7,014	6,765	-250	(-4,431, 3,932)	1
**Heterophils**	**/μL**	20	1,205	1,873	668	(-3,361, 4,697)	1
		25.6	772	900	29	(-2,952, 3,009)	1
**Lymphocytes**	**/μL**	20	2,832	4,916	2,084	(290, 3,877)	0.01
		25.6	2,227	2,559	332	(-995, 1,658)	1
**Monocytes**	**/μL**	20	1,111	828	-282	(-823, 258)	0.7
		25.6	831	409	-422	(-870, 27)	0.08
**Azurophils**	**/μL**	20	1,730	3,121	1,391	(93, 2,690)	0.03
		25.6	1,834	3,226	1,391	(93, 2,690)	0.03
**Basophils**	**/μL**	20	19	6	-13	(-33, 6)	0.4
		25.6	38	25	-13	(-33, 6)	0.4
**Heterophils/ Lymphocytes**	** **	20	1.148	0.962	-0.186	(-7.63, 7.25)	1
		25.6	0.531	0.354	-0.177	(-5.68, 5.33)	1
**Analyte**	**Units**	**Temp (** ^**o**^ **C)**	**Mean Study Day 0**	**Mean Study Day 49**	**Difference**	**95% Confidence Interval**	**P-value**
**PCV**	**%**	20	42.7	34.6	-8.0	(-11.6, -4.5)	< 0.0001
		25.6	41.9	37.5	-4.5	(-7.1, -1.8)	< 0.0001
**TS**	**g/dL**	20	8.8	9.1	0.3	(-0.4, 1.1)	0.8
		25.6	9.3	8.5	-0.8	(-1.3, -0.3)	0.0004
**WBC**	**/μL**	20	6,831	10,737	3,906	(-1,137, 8,948)	0.3
		25.6	7,014	6,765	-250	(-4,431, 3,932)	1
**Heterophils**	**/μL**	20	1,205	1,873	668	(-3,361, 4,697)	1
		25.6	772	900	29	(-2,952, 3,009)	1
**Lymphocytes**	**/μL**	20	2,832	4,916	2,084	(290, 3,877)	0.01
		25.6	2,227	2,559	332	(-995, 1,658)	1
**Monocytes**	**/μL**	20	1,111	828	-282	(-823, 258)	0.7
		25.6	831	409	-422	(-870, 27)	0.08
**Azurophils**	**/μL**	20	1,730	3,121	1,391	(93, 2,690)	0.03
		25.6	1,834	3,226	1,391	(93, 2,690)	0.03
**Basophils**	**/μL**	20	19	6	-13	(-33, 6)	0.4
		25.6	38	25	-13	(-33, 6)	0.4
**Heterophils/ Lymphocytes**	** **	20	1.148	0.962	-0.186	(-7.63, 7.25)	1
		25.6	0.531	0.354	-0.177	(-5.68, 5.33)	1

Snakes inoculated with *O*. *ophidiicola* had pronounced hematologic changes at 20°C with less severe alterations at 26°C. Snakes maintained at the cooler temperature had significant increases in WBC, heterophil count, azurophil count, and the H:L over time (Table 3). Snakes in the warmer temperature had increases in WBC and azurophil count and a decrease in PCV (Table 3).

**Table 3:** Differences in inoculated prairie rattlesnake (*Crotalus viridis*) hematologic parameters between study day 49 (last day all snakes remained alive) and study day 0 (date of inoculation with *Ophidiomyces ophidiicola* or sterile saline injection) at two different ambient temperatures. Day 0 is the reference value.

Analyte	Units	Temp (°C)	Mean Study Day 0	Mean Study Day 49	Difference	95% Confidence Interval	P-value
**PCV**	**%**	20	37.6	35.9	-1.7	(-4.8, 1.4)	0.7
		25.6	31.3	26.5	-4.7	(-7.1, -2.3)	< 0.0001
**TS**	**g/dL**	20	7.9	8.3	0.4	(-0.3, 1.0)	0.6
		25.6	8	8	0.05	(-0.4, 0.5)	1
**WBC**	**/μL**	20	7,817	16,049	8,233	(3,587, 12,878)	< 0.0001
		25.6	8,000	12,077	4,078	(208, 7,947)	0.03
**Heterophils**	**/μL**	20	2,045	8,258	6,213	(2,747, 9,679)	< 0.0001
		25.6	1,707	2,640	933	(-1,757, 3,623)	1
**Lymphocytes**	**/μL**	20	1,010	1,057	47	(-1,497, 1,590)	1
		25.6	2,255	2,956	702	(-497, 1,901)	0.6
**Monocytes**	**/μL**	20	725	891	166	(-332, 664)	1
		25.6	446	472	26	(-388, 441)	1
**Azurophils**	**/μL**	20	4,207	5,598	1,391	(93, 2,690)	0.03
		25.6	4,311	5,703	1,391	(93, 2,690)	0.03
**Basophils**	**/μL**	20	15	1	-13	(-33, 6)	0.4
		25.6	33	20	-13	(-33, 6)	0.4
**Heterophils/ Lymphocytes**	** **	20	2.693	13.23	10.535	(4.14, 16.93)	< 0.0001
		25.6	0.849	1.067	0.218	(-4.74, 5.18)	1

On day 49 pi, the last date where all snakes remained alive, inoculated snakes at 20°C had higher heterophil counts and H:L and lower lymphocyte counts compared to control snakes (Table 4). On the same day, inoculated snakes at 20°C had significantly higher heterophil counts and H:L compared to both inoculated (heterophil effect size = 5,618 cells/μL, 95% confidence interval = 1,210–10,026 cells/μL, p = 0.01; H:L effect size = 12.16, 95% confidence interval = 5.06–19.26, p = 0.001) and control snakes (heterophil effect size = 7,458 cells/μL, 95% confidence interval = 2,524–12,392 cells/μL, p = 0.005; H:L effect size = 12.87, 95% confidence interval = 4.99–20.76, p = 0.003) at 26°C.

**Table 4:** Differences in prairie rattlesnake (*Crotalus viridis*) hematologic parameters between snakes inoculated with *Ophidiomyces ophidiicola* and control snakes injected with sterile saline on study day 49 (last day all snakes remained alive) at two different ambient temperatures. Control snakes are the reference value.

Analyte	Units	Temp (°C)	Control Mean	Inoculated Mean	Difference	95% Confidence Interval	P-value
**PCV**	**%**	20	34.6	35.9	1.3	(-13.4, 16.0)	1
		25.6	37.5	26.5	-11.0	(-26.5, 4.6)	0.2
**TS**	**g/dL**	20	9.1	8.3	-1.1	(-6.7, 4.5)	1
		25.6	8.5	8	-0.5	(-6.1, 5.1)	1
**WBC**	**/μL**	20	10,737	16,049	5,313	(-2,987, 13,612)	0.3
		25.6	6,765	12,077	5,313	(-2,987, 13,612)	0.3
**Heterophils**	**/μL**	20	1,873	8,258	6,386	(247, 12,524)	0.04
		25.6	900	2,640	1,840	(-4,617, 8,297)	0.9
**Lymphocytes**	**/μL**	20	4,916	1,057	-3,859	(-7,292, -427)	0.03
		25.6	2,559	2,956	397	(-3,242, 4,035)	1
**Monocytes**	**/μL**	20	828	891	63	(-758, 883)	1
		25.6	409	472	63	(-758, 883)	1
**Azurophils**	**/μL**	20	3,121	5,598	2,477	(-2,452, 7,406)	0.5
		25.6	3,226	5,703	2,477	(-2,452, 7,406)	0.5
**Basophils**	**/μL**	20	6	1	-5	(-50, 41)	1
		25.6	25	20	-5	(-50, 41)	1
**Heterophils/ Lymphocytes**	** **	20	0.962	13.23	12.266	(2.38, 22.16)	0.01
		25.6	0.354	1.067	0.714	(-9.58, 11.01)	1

## Histopathology

**Fig 9:**
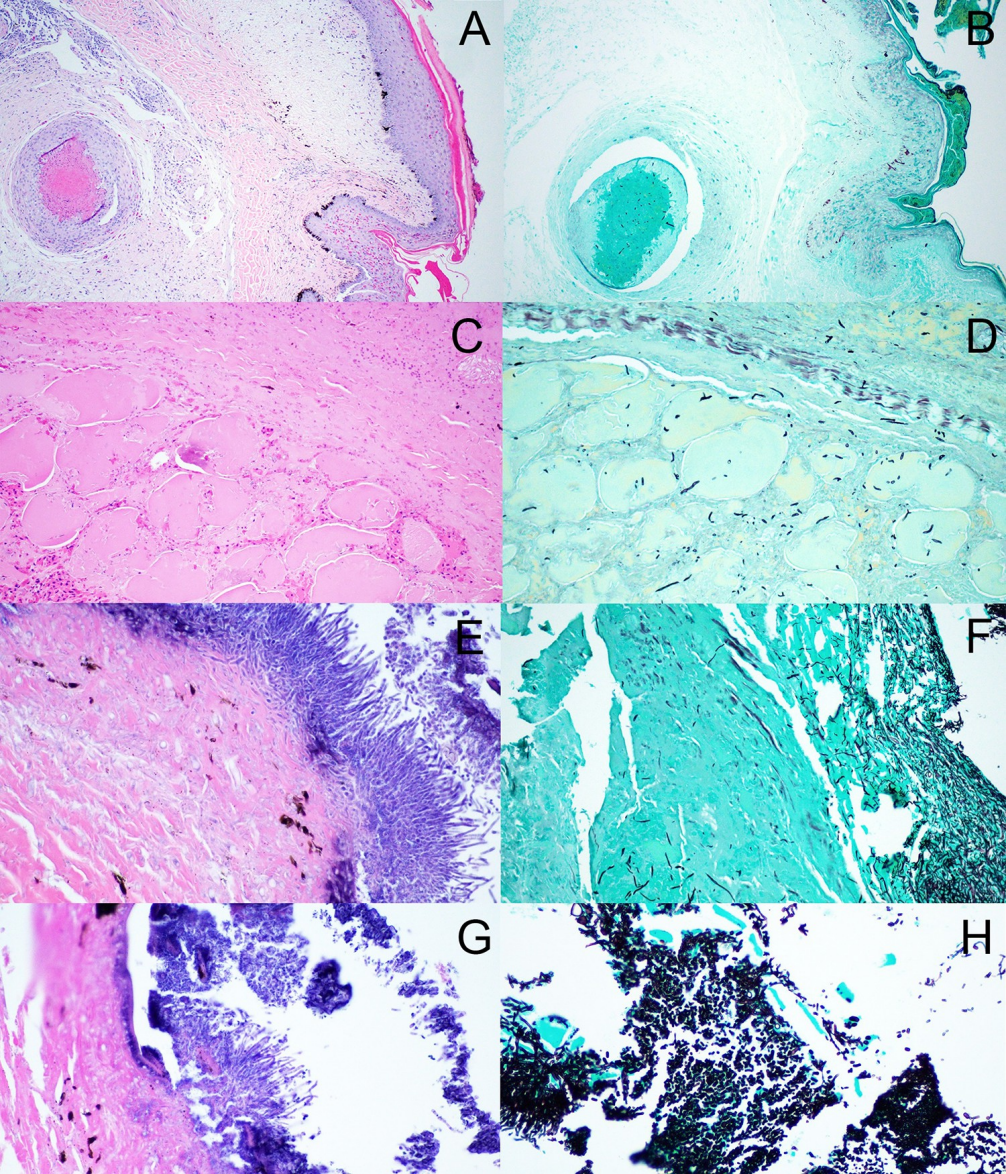
Histopathology of Ophidiomyces in Prairie rattlesnakes. Histologic lesions in prairie rattlesnakes (*Crotalus viridis*) experimentally challenged with *Ophidiomyces ophidiicola*. [A/B], Skin and subcutis from 26°C inoculated snake. A) The epidermis is moderately to markedly hyperplastic with abundant serocellular crusts and the subcutis has several large granulomas (hematoxylin and eosin (HE, 20x); B) Gomori’s methenamine silver (GMS) staining highlights fungal hyphae in the crusts, epidermis, and granulomas. [C-H], Subcutis and skeletal muscle from 20°C inoculated snake. C) There is extensive necrosis and hemorrhage in the subcutis and skeletal muscle with mild inflammation (HE, 20x); D) GMS staining highlights fungal hyphae within necrotic tissues. E/F, Skin, ulcer from 20°C inoculated snake. E) The epidermis is absent (ulceration) and the exposed dermis is smudged and hypereosinophilic (degeneration). The surface is overlain by thick mat of fungi including superficial arthroconidia and fungal hyphae in dermis and deeper tissues (HE, 20x). F) GMS staining shows unfettered fungal invasion. G) Superficial fungal mats have cylindrical arthroconidia (*) at the air surface (HE, 60x); H) Superficial fungal mats with GMS staining (HE, 60x).

Histologic lesions were found primarily in the skin of all inoculated snakes and generally differentiated into two distinct disease patterns. The inoculated snakes housed at 20°C (n = 4) had regionally extensive, severe cutaneous disease with complete necrosis and loss (ulceration) of the epidermis, and degeneration and necrosis of the dermis, subcutis and superficial to deep skeletal muscle (Fig 9C and 9D). These lesions contained low numbers of inflammatory cells, myriad fungi present in mats superficially together with bacterial aggregates, and fungi in deeper tissues, indicative of invasion (Fig 9C–9H). The inoculated snakes housed at 26°C (n = 3) had proliferative and inflammatory cutaneous disease with marked epidermal hyperplasia, intraepithelial heterophils, severe hyperkeratosis and serocellular crusting, and overall mild, multifocal epidermal necrosis, erosion, and ulceration (Fig 9A and 9B). The 26°C inoculated snakes also had robust granulomatous cellulitis with numerous large nodular accumulations of necrotic debris and peripheral bands of epithelioid macrophages and multinucleated giant cells. Many granulomas contained low numbers of central fungal hyphae. One control snake in the 20°C group had focal heterophilic and proliferative epidermitis with rare intraepidermal fungi without invasion, while the other 20°C group control snakes had normal skin. One control snake in the 26°C group had focal epidermal hyperplasia without necrosis, inflammation, or fungi, the other 26°C group control snake had normal skin.

Overall, 20°C group snakes had the most severe lesions with minimal inflammatory response and evidence of deep fungal invasion. Two individuals in the 20°C group had fungal dissemination evidenced by granulomas in several parenchymal organs and coelomic connective tissues with positive GMS staining. One of these snakes also had multisystemic bacterial infection affecting the vertebral spine, spinal cord, and associated skeletal muscle. In contrast, one 26°C inoculated snake had a focal hepatic GMS-positive granuloma. Intralesional fungi were characteristic of *O*. *ophidiicola*: up to 5 micron diameter, parallel-walled hyphae with occasional septations and non-dichotomous branching. Within superficial fungal mats and in outer layers of the serocellular crusts (at air interface) were variable numbers of cylindrical arthroconidia.

## Discussion

This project set out to characterize the host response to an experimental inoculation with *Ophidiomyces ophidiicola* in prairie rattlesnakes at two environmental temperatures within the species’ preferred optimum temperature zone [51]. A novel and effective intradermal method for inducing ophidiomycosis is presented that can be used in future challenge studies. Previous methods for inoculation have included application of pure fungal culture directly to nasolabial pits [6] or placing conidial suspension on a bandage, then setting the bandage over several sites on the snake’s body [4,52]. The latter method had greater success forming a gross lesion if the site was abraded prior to inoculation [4]. All three studies indicate that a compromised epidermis plays an important role in the development of apparent ophidiomycosis. The intradermal inoculation method used by the present study bypassed the epidermis entirely by injecting fungal culture directly into the dermis, therefore circumventing any natural existing barrier to the fungus. Compared to other published inoculation methods, our approach is less likely to mimic natural routes of skin colonization and infection with *O*. *ophidiicola*. However, methods that abrade skin create more disease variability as irritation of the skin may allow other opportunistic infections to occur and may be less repeatable due to the inherent subjective nature of pressure applied during the process. This was supported in this study as all snakes that had epidermal defects had opportunistic bacterial colonization, even control snakes. Additionally, unlike previous studies, no snake in this study had to be re-inoculated and shedding did not relieve snakes of lesions. Ophidiomycosis has been identified in numerous Serpentes taxa, many with different scale types and cutaneous microbiota [12,53]. Routes of natural transmission are poorly characterized but are proposed to occur through direct contact with infected soil or other snakes [54,55], a process which is likely affected by scale type, skin microbiome, and natural history of each species [56,57]. Thus, until mechanisms for natural transmission are clarified, intradermal inoculation provides a highly repeatable method for observing the effects of this disease in a host.

In this study, survival time was significantly different between control and inoculated groups as expected, which was largely driven by 100% mortality in the 20°C group. Previous studies have shown variable mortality rates after experimental inoculation at temperatures close to 26°C [6], thus the 33% mortality rate in the 26°C inoculated group in the present study is not surprising. The MST of 62 days in the 20°C group is considerably different from the previously documented 90 days in cottonmouths (*Agkistrodon piscivorus*) maintained at temperatures close to 26°C [6]. Although all the inoculated 20°C snakes were euthanized prior to the study end date, it is important to note that the severity of their lesions histologically and gross clinical presentation indicated they were unlikely to improve or recover with more time. The higher mortality rate at lower temperatures may explain why the development of skin lesions consistent with ophidiomycosis is observed more commonly during cooler months in free-living snakes [58,59], and possibly why some animals recover as temperatures increase. Furthermore, it is possible that seasonal differences may account for temporal variation seen in the wild, but as this study was performed indoors starting in December, it seems unlikely that seasonal differences alone accounted for the development of disease.

Several key differences in behavioral parameters were observed between control and inoculated snakes at both environmental temperatures, highlighting that the snake response to the fungal pathogen, or the fungus itself, behaves differently based on temperature. The increase in eating, defecating, and shedding in the 26°C group are likely associated with the increased metabolic rate of snakes at higher temperatures [31,56,60]. Whereas the significant changes in activity levels and time outside of the hidebox observed between control and inoculated snakes are likely associated with the host response to infection and were further modulated by temperature. Previous reports in free-ranging rattlesnakes have observed longer basking times in infected snakes [8] and support the results of the current study. This finding is likely related to behavioral fever demonstrated by numerous ectotherms [35]. However, the 20°C group spending more time in the hidebox may be masking behavioral changes the inoculated snakes would be more likely to exhibit in a warmer environment. Exploring the nuances of behavioral change in response to disease in reptiles has always posed a challenge for many reasons including its apparent subtlety to the observer, differences in field and captive behavior, and variability of behavior across vast taxa [61,62]. The behavioral changes in this study noted between temperatures, not just treatment status alone, emphasize the caveats of relying solely on behavioral indicators to detect illness in reptiles without considering other impactful variables such as temperature.

Clinical signs were significantly associated with inoculation, consistent with previous challenge study experiments [4,6,52], and in free-ranging snakes with *O*. *ophidiicola* qPCR detection [13,14,19,40,63]. The most common lesion type initially observed through physical examination was a displaced scale, regardless of temperature. This seems logical but is rarely a sign observed in natural infections of free-ranging snakes where other lesion types are more common [5,20,34]. This is likely due to the fact that free-ranging snakes are sampled at unknown times during disease development and are rarely identified in the early stages. Lesion progression in the present study was similar to others [4] with lesions transitioning from displaced scales to necrosis to multiple adjacent infected/necrotic cells and/or sloughing of infected scales leading to ulcers. In addition to gross observation, the lesions were evaluated with UV fluorescence and fungal copy number. Visual images of lesions under UV light compared to corresponding natural light images showed distinct progression of focal scale abnormalities to large coalescing lesions. Unfortunately, UV fluorescence has a lower sensitivity and specificity than the presence of clinical signs at the same qPCR detection threshold, which is attributed to the UV fluorescence observed in control snakes. UV fluorescence has been observed to be non-specific for changes in keratinized tissue and normal physiologic processes or external contamination with exogenous substances can cause a positive result [64]. Despite this progression in appearance and presumed severity, no significant differences were observed in fungal copy number between the gross lesion types. Previously, ulcers were reported to have the lowest fungal copy number compared to all other lesions, presumably associated with a decrease in keratinized epidermal cells in those lesions given that *O*. *ophidiicola* is keratinophilic [5], however that was not observed in this study. In the current study, *O*. *ophidiicola* qPCR copy number standardized based on the DNA concentration of body swabs was compared in snakes with different lesion classifications rather than the non-standardized qPCR copy numbers from skin lesion swabs, likely causing the difference in results seen from the previous study. The lack of difference in lesion type, lesion number, and fungal copy number between inoculated snakes at both temperatures likely supports that the process by which *O*. *ophidiicola* infects snakes is temperature-independent, but the outcome (length of survival), which is at least partially mediated by host response (e.g. hematologic and histologic changes), is temperature-dependent.

Hematology is a common veterinary diagnostic used to assess a patient’s response at an isolated point and over time. In reptiles, the hematologic response often varies by season [30,65], temperature [32], species [24,25], sex [23,66], and age class [67], but rarely have studies been performed looking at specific disease states, and those are often observational [21]. The changes seen in prairie rattlesnakes in this study were complex, but frequently included differences over time, between treatments, and at both temperatures regardless of sex. The total WBC count is a measure of systemic inflammation, and it increased in inoculated snakes at both temperatures (although the degree was greater in snakes at 20°C) over time, largely driven by concurrent increases in heterophils in the 20°C group and azurophils at 26°C. Heterophils are phagocytic cells that play a key role in the inflammatory response of many reptiles’ immune systems [68,69]. Heterophilia is generally associated with active inflammation that may be nonspecific or due to infectious causes [26,28]. Azurophils are phagocytic and elevations are typically associated with inflammation, but relative percentages and classification of these cells differ depending on the species [26]. In many snakes, including viperids, they are counted as their own cell category, are considered to function similarly to mammalian neutrophils, but morphologically contain features of both monocytes and granulocytes [28]. Previous studies have also shown increased heterophils and azurophils in *Sistrurus catenatus* sampled at a single time point during their ophidiomycosis disease process compared to snakes without ophidiomycosis [20]. A relative azurophilia was noted in a previous study in *Nerodia sipedon insularum* that had skin lesions and were *O*. *ophidiicola* positive compared to those that were *O*. *ophidiicola* negative or positive with no skin lesions [70]. However, the control snakes in this study also had significant increases in azurophils over time at both temperatures, making azurophilia a less compelling indicator of response to *O*. *ophidiicola*. Heterophil to lymphocyte ratio (H:L) is commonly utilized as a conserved measure of physiologic stress in reptiles [71]. The significantly elevated H:L in the inoculated snakes kept at 20°C demonstrates that this group was more heavily impacted by infection than the inoculated snakes kept at 26°C. This is further supported by the much larger elevation in heterophils in this group, suggesting a more robust immune response to *O*. *ophidiicola* infection.

The histopathologic lesions supported the progression of disease described above as snakes in the 26°C group had moderate to marked epidermal proliferative changes with intense inflammation centered on fungi, and lesser necrosis and ulceration. In contrast, snakes in the 20°C group had extensive epidermal ulceration, severe transmural (cutaneous and body wall) necrosis with minimal inflammation and seemingly unencumbered fungal proliferation and dissemination. In a previous study that included histopathologic examinations of ophidiomycosis lesions [72], histologic grades were often determined by the number and type of inflammatory cell components in tissues, depth of lesion invasion, and presence of necrosis, among other factors. The same study also proposed that decreased inflammatory cells are associated with late stage or chronic lesions in addition to acute infection. In the present study, the 20°C group had more severe lesions on histopathologic evaluation based on evidence of deep invasion and necrosis, however, the presence of inflammatory cells associated with these lesions was significantly reduced compared to inoculated snakes at 26°C, suggestive of more chronic lesions. Inoculated snakes in the 20°C group died significantly earlier than those in the 26°C group. Thus, when their lesions were examined, one would expect those of the 26°C group to be considerably more chronic in appearance since lesion development occurred on a similar timeline post-inoculation and these individuals survived longer. However, it was the 20°C group that presented with more necrosis, deeper infiltration, and less overall inflammation. This suggests that time alone does not determine severity of lesions and that lesion development at the tissue level occurs more rapidly at the cooler temperature which leads to more advanced, chronic lesions in a shorter timeframe. Importantly, in vitro *O*. *ophidiicola* growth is significantly slower at 20°C compared to 25°C [54], so more aggressive temperature-driven fungal replication is an unlikely explanation for these findings.

It is worth noting that tissue inflammation was more prominent in the 26°C group than the 20°C group while the hematology findings seem to indicate the opposite. This is an example of how circulating inflammatory cell response does not always correlate with what is occurring at the tissue level. Sites of insult in poultry have been demonstrated to recruit heterophils in a chemotactic mechanism that utilizes cytokines similar to IL-8 [73]. Neutrophils, considered the mammalian counterpart to heterophils [74], have been shown to migrate more effectively at elevated temperatures [75]. Although the 26°C group appeared to have fewer inflammatory cells in peripheral circulation, the vigorous inflammatory response seen in the affected tissue suggests these snakes had no difficulty recruiting heterophils to the site of infection. The results of this study bolster the notion that ambient temperature plays a role in the ectotherm immune response as well as the outcome of disease.

Previous studies have demonstrated negative associations between environmental temperature, body condition, and *O*. *ophidiicola* infection severity in free-living pigmy rattlesnakes (*Sistrurus miliarius*) [10,76]. Plasma corticosterone (CORT) concentrations are also negatively associated with temperature and body condition in this species, with peak CORT concentrations occurring in winter months when ophidiomycosis lesions are most severe [10]. These associations are hypothesized to be due to a seasonal interplay between environmental factors, host energetics, and the hypothalamo-pituitary-adrenal axis which drive a positive feedback loop involving increased allostatic load, worsening host condition, and increasing ophidiomycosis severity during winter months. It is specifically posited that environmental factors such as temperature and food availability drive negative energy balance, initiating a cascade of negative physiologic effects which increase host susceptibility to *O*. *ophidiicola* infection. Our findings challenge this hypothesis, as inoculated snakes did not experience significant weight loss or body condition changes during the course of the study. Instead, our findings indicate that environmental temperature changes, independent of alterations in energy balance, are enough to significantly alter host response to infection. It is highly likely that decreased resource availability exacerbates temperature-associated effects in free-living snakes, but our findings indicate an independent role for these physiologic drivers. This underscores the inherent complexity of the host-pathogen-environmental relationship in ectothermic vertebrates and highlights the utility of controlled experimental studies to disentangle causal relationships.

Limitations in this study include the small sample size to evaluate many continuous variables (disease severity, absolute leukocytes, mass). Due to animal welfare and use concerns, the sample size was determined for the most important hypothesis in this study, which was survival differences of the snakes. Future studies that evaluate subsequent more subtle hypotheses, such as impact of inoculation dose, changes in lesion severity, and hematologic responses should be performed with larger sample size utilizing data in this study as *a priori* information. Inferences made about the immune function of snakes in this study were made solely on hematologic data. These interpretations would be better supported and described via supplementation with other modalities such as cytokine investigation and transcriptomics.

In conclusion, this study described several behavioral, clinical, molecular, hematological, and histopathological findings in prairie rattlesnakes experimentally challenged with *Ophidiomyces ophidiicola*. Findings demonstrate that temperature had a profound impact on disease progression and that future investigations should target specific areas of the host response to better understand the disease process. Identifying the drivers of infection is crucial to implementing management or therapeutic strategies that minimize the impact of this disease. Ophidiomycosis remains a significant disease to certain species and populations, and the results of this study confirm that at least some of the variability in disease outcomes is associated with environmental temperature.

## Supporting information

S1 Fig Photograph of intradermal injection. Intradermal injection into a single scale of a Prairie rattlesnake (Crotalus viridis) with either Ophidiomyces ophidiicola or sterile saline.
(TIF)

S1 Raw data (XLSX)
