# Supplementary figures and images for: Environmental temperature influences ophidiomycosis progression and survival in experimentally challenged prairie rattlesnakes (Crotalus viridis)
Source: PLoS One. 2023 Aug 3;18(8):e0289641. doi: 10.1371/journal.pone.0289641 (PMC10399908; doi:10.1371/journal.pone.0289641)

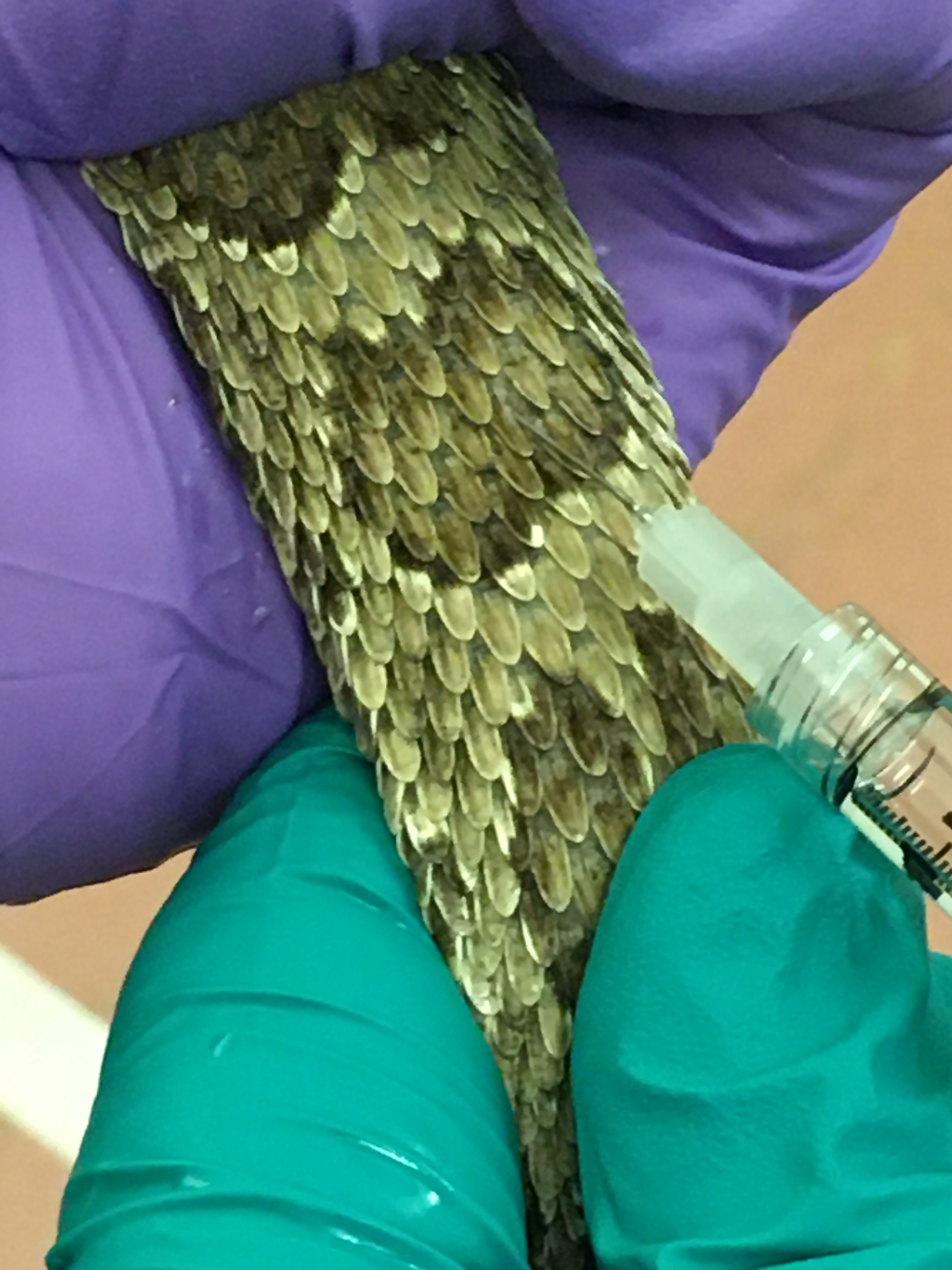

Supplement: S1 Fig — Intradermal injection into a single scale of a Prairie rattlesnake (Crotalus viridis) with either Ophidiomyces ophidiicola or sterile saline. (TIF) [file pone.0289641.s001.tif]
